# Immunogenicity and contraceptive efficacy of plant-produced putative mouse-specific contraceptive peptides

**DOI:** 10.3389/fpls.2023.1191640

**Published:** 2023-06-28

**Authors:** Khadijeh Ghasemian, Inge Broer, Jennifer Schön, Nadine Kolp, Richard Killisch, Stefan Mikkat, Jana Huckauf

**Affiliations:** ^1^ Department of Agrobiotechnology and Risk Assessment for Bio and Gene Technology, Faculty of Agricultural and Environmental Sciences, University of Rostock, Rostock, Germany; ^2^ Department of Reproduction Biology, Leibniz Institute for Zoo and Wildlife Research (IZW), Berlin, Germany; ^3^ BIOSERV, Analytik und Medizinprodukte GmbH, Rostock, Germany; ^4^ Core Facility Proteome Analysis, Rostock University Medical Center, Rostock, Germany

**Keywords:** plant molecular farming, *Nicotiana benthamiana*, contraceptive vaccine, Zona pellucida, Izumo

## Abstract

Rodent population control through contraception requires species-specific oral contraceptive vaccines. Therefore, in this study, we produced putative mouse-specific contraceptive peptides, mZP2 (from oocyte) and mIzumo1 (from sperm), in plants using *Agrobacterium*-mediated transient expression. Peptides were produced separately in *Nicotiana benthamiana* using constructs encoding antigens containing three copies of each peptide. We also determined the immunogenicity and contraceptive effects of the plant-produced antigens in female BALB/c mice. Mice immunized subcutaneously with a relatively low amount of antigen (5 µg/dose of each peptide in a mixture) showed systemic immune responses against mZP2-3 and mIzumo1-3 antigens. Moreover, the mean litter size of mice treated with the plant-produced antigens was reduced by 39% compared to that of the control mice. Notably, there was a significant negative correlation between the number of pups born and individual antibody levels against both antigens. Immunofluorescence assays demonstrated the binding of induced antibodies to the oocytes of BALB/c and wild-type mice *in vivo* and *in vitro*, respectively. Our study demonstrate the feasibility of producing small contraceptive peptides in plants that can be further used to develop oral contraceptive vaccines against mouse populations.

## 1 Introduction

Increased population of rodent pests, such as mice, causes economic and environmental damage worldwide (Singleton et al., 2010; Wondifraw et al., 2021; Brown and Henry, 2022). Therefore, effective population management strategies are needed to control these pests (Jacob, 2008; Jacoblinnert et al., 2021). Fertility control is considered a safer alternative to currently used methods, such as culling, trapping, and poisoning, which are unethical, stimulate reproduction, and pose a risk to non-target species (Barfield et al., 2006; Singleton et al., 2007; Imakando et al., 2021). Immunocontraception is the use of vaccines to reduce fertility by inducing immune responses against essential proteins involved in various stages of fertilization Chambers et al., 1997; Barber and Fayrer-Hosken, 2000; Naz, 2005). Several egg- and sperm-specific candidate antigens capable of inhibiting fertility have been identified [reviewed in (Naz, 2005; Gupta and Minhas, 2017)].

Proteins of Zona pellucida (ZP), an extracellular glycoprotein matrix covering the mammalian oocytes, are most commonly used as contraceptive vaccine antigens (Barber and Fayrer-Hosken, 2000; Gupta et al., 2004). ZP2, the secondary receptor of sperm, is an essential protein for reproduction (Bleil et al., 1988; Wassarman, 1988), and antibodies against its epitopes interfere with sperm-binding and inhibit fertilization in mice (Hinsch et al., 1998; Sun, 1999; Hardy et al., 2008). In addition to oocyte-specific antigens, several spermatozoa-specific antigens have been proposed as targets for immunocontraception. Izumo1 is a sperm-specific protein localized in the acrosomal region of sperm that plays a vital role in sperm–egg fusion and fertilization (Inoue et al., 2005; Naz, 2014; Xue et al., 2016). A previous study demonstrated that sperms produced by Izumo1-deficient mice are unable to fuse with eggs (Inoue et al., 2005). Izumo1 is exposed and accessible in female reproductive tract after the acrosome reaction (Inoue et al., 2005). Izumo1 can induce immune responses in the serum and genital tracts of immunized mice (Naz, 2008). Immunization with vaccines based on mouse Izumo1 significantly decreases the fertility and litter size of mice (Naz, 2008; An et al., 2009; Wang et al., 2009).

Peptide-based contraceptive immunogens can improve the safety and species-specificity of vaccines (Hinsch et al., 1999; Ferro and Mordini, 2004; Hardy et al., 2004), which are crucial for vaccines intended to target wildlife populations (Bradley et al., 1999; Kirkpatrick et al., 2011). ZP proteins are not species-specific (Frank et al., 2005; Naz, 2005); therefore, antigens must be reduced to the species-specific regions of the oocyte and sperm proteins. Targeting peptides from functional and less conserved regions of contraceptive proteins may be a promising approach to achieve species-specificity (Lou et al., 1995; Hinsch et al., 1999; Hardy et al., 2008). Contraceptive and putative mouse-specific peptides, mZP2 (Sun, 1999; Hardy et al., 2008) and mIzumo1 (Naz, 2008; Xue et al., 2016), are effective in reducing the fertility of female mice. Peptide vaccines are less effective compared with protein vaccines (Ferro and Mordini, 2004). However, incorporation of adjuvants and multiple epitopes into antigens can improve the immunogenicity and efficacy of peptide-based antigens (Sadler et al., 2002; Ferro and Mordini, 2004; Hardy et al., 2004; Ghasemian et al., 2023a). Production of peptide-based mouse-specific contraceptive antigens facilitates the oral administration of vaccines to widely dispersed animals.

Transgenic plants expressing immunocontraceptive vaccines provide a safe production and delivery system. In addition to their simple production and easy scale-up processes, plant expression systems present a minimal risk of toxicity and contamination with mammalian pathogens (Daniell et al., 2009; Kwon et al., 2013). Use of plant-based contraceptive vaccines provides a relatively cost-effective control strategy to target free-ranging wildlife species (Smith et al., 1997; Polkinghorne et al., 2005). These vaccines can be further formulated as oral baits or used directly as feed (Daniell et al., 2009), enabling the widespread application of contraceptives for mouse population management.

In this study, we developed a proof-of-principle for the use of plants as systems for the production of contraceptive peptides. We used a transient expression system (viral-based magnICON system) to produce mZP2 and mIzumo1 peptides, from mouse ZP2 and Izumo1 proteins, respectively, in *Nicotiana benthamiana*. We also assessed the immunogenicity of plant-produced antigens in immunized mice as well as the fertility of vaccinated mice. Moreover, immunohistochemistry was used to investigate the *in vivo* and *in vitro* reactivities of the induced antibodies.

## 2 Materials and methods

### 2.1 Construction of plant expression vectors

**Figure 1:**
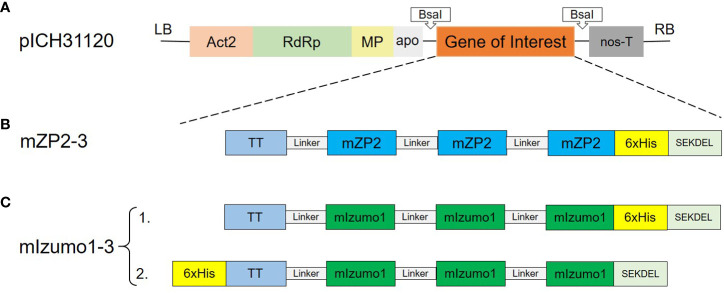
Schematic representation of the T-DNA region of the MagnICON vector and expression constructs used in this study. **(A)** Binary pICH31120 expression vector. Act2, *Arabidopsis* actin 2 promoter; RdRp, RNA-dependent RNA polymerase; MP, tobacco mosaic virus (TMV) movement protein; apo, apple pectinase-apoplastidal targeting sequence; nos-T, *Agrobacterium tumefaciens* nopaline synthetase terminator; LB, left border; RB, right border. **(B)** Construct used for the expression of mouse ZP2 peptide. **(C)** Construct used for the expression of mouse Izumo1 peptide. TT, T-cell epitope of tetanus toxoid (aa residues 830–844); mZP2, T- and B-cell epitopes of mouse ZP2 (aa residues 121–139); mIzumo1, B-cell epitope of mouse Izumo1 (aa residues 163–183); Linker; (GGGGS)3 flexible linker; 6xHis, Hexa histidine tag. SEKDEL: ER retrieval peptide.

Coding sequences of mZP2-3 (containing three copies of mZP2 peptide, TDVRYKDDMYHFFCPAIQA) and mIzumo1-3 (containing three copies of mIzumo1 peptide, LDCGERHIEVHRSEDLVLDCL) constructs (**Figure 1**) were designed with optimized codons for expression in *N. benthamiana.* Gene constructs were synthesized by Eurofins Genomics GmbH (Ebersberg, Germany) and cloned into the pEX-A vector. Coding regions were flanked by BsaI restriction sites after polymerase chain reaction (PCR) amplification. PCR products were cloned separately into the pJET1.2 cloning vector (CloneJET PCR Cloning Kit; Thermo Fisher Scientific, Waltham, Massachusetts, USA). Coding sequences were cloned into a tobacco mosaic virus (TMV)-based expression vector of the MagnICON transient expression system, pICH31120, with BsaI restriction and ligation (Engler et al., 2008). MagnICON vectors were kindly provided by Nomad Bioscience GmbH (Halle/Saale, Germany).

### 2.2 Transient expression of mZP2-3 and mIzumo1-3 in *N. benthamiana* leaves

Recombinant expression vectors were separately introduced into *Agrobacterium tumefaciens* strain ICF320 via electroporation. The resulting transformed colonies (confirmed using colony PCR and restriction enzyme digestion) were inoculated into 5 mL of Luria–Bertani (LB) medium supplemented with 50 μg/mL kanamycin and 50 μg/mL rifampicin and incubated overnight at 28°C with shaking (220 rpm). To prepare the main bacterial culture, 2 mL of the *Agrobacterium* suspension was placed in 200 mL LB medium containing the same antibiotics and incubated overnight (220 rpm, 28°C). *Agrobacterium* cells were harvested at 4560 × *g* for 30 min and resuspended in the infiltration medium (10 mM 2-(N-morpholino) ethanesulfonic acid [pH 5.8], 10 mM MgSO_4_, and 0.02% v/v Silwet Gold) to achieve a final OD_600_ of 0.15–0.2. Bacterial suspension was then vacuum-agroinfiltrated into the leaves of 6–8-week-old greenhouse-grown *N. benthamiana* plants as previously described (Nausch and Broer, 2017).

### 2.3 Total soluble protein extraction from *N. benthamiana* leaves

Agroinfiltrated *N. benthamiana* leaves were harvested at various days post-infiltration (dpi), frozen at −80 °C, lyophilized using a freeze dryer system (Alpha 1–4 LD; Martin Christ GmbH, Osterode, Germany), and pulverized using a mixer. Small-scale protein extraction was performed to evaluate the protein expression and accumulation. Total soluble protein (TSP) was extracted from 25 mg of lyophilized leaf material, homogenized in 500 µL cold protein extraction buffer (100 mM NaCl, 10 mM KCl, 6.5 mM Na_2_HPO_4_, 2 mM KH_2_PO_4_, pH 7.2) plus complete protease inhibitor cocktail (Roche Diagnostics GmbH, Mannheim, Germany), using a Precellys 24 homogenizer (Bertin Instruments, France), and incubated for 30 min on ice. Clarified extract was obtained via repeated centrifugation at 15000 × *g* for 15 min at 4°C. Then, protein concentration of the supernatant was measured using the Pierce Coomassie Plus (Bradford) assay kit (Thermo Fisher Scientific) with bovine serum albumin (BSA) (Thermo Fisher Scientific) as the standard.

### 2.4 Enrichment of proteins using the Ni-NTA purification system

Histidine (His)-tagged recombinant mZP2-3 and mIzumo1-3 were partially purified via immobilized metal affinity chromatography (IMAC) using a Ni-chelating resin. Leaf extracts (in 50 mM NaH_2_PO_4_, 300 mM NaCl, 250 mM sucrose, 5 mM imidazole) were centrifuged at 16,000 × *g* and 4°C for 1 h. Supernatants were collected and loaded onto a column (Bio-Rad Laboratories, Hercules, California, USA) containing pre-equilibrated Nuvia IMAC Ni-charged resin (Bio-Rad Laboratories). The column was washed twice with the wash buffer containing 20 and 30 mM imidazole. Recombinant protein was eluted with an elution buffer containing 300 mM imidazole, and the elution fraction was concentrated and desalted using Vivaspin 20 centrifugal concentrators with a 10 kDa cut-off membrane (Sartorius AG, Germany). LC-MS analyses of partially purified mIzumo1-3 protein were performed as already described (Klähn et al., 2021).

### 2.5 Sodium dodecyl sulfate-polyacrylamide gel electrophoresis (SDS-PAGE) and western blotting analysis

Protein samples were denatured at 95°C for 5 min in a loading buffer (10% glycerin, 150 mM Tris [pH 6.8], 3% SDS, 1% β-mercaptoethanol, and 2.5% bromophenol blue) and subjected to 12% SDS-PAGE. The separated proteins were transferred onto a 0.45-μm Hybond ECL nitrocellulose membrane (GE Healthcare Europe GmbH, Freiburg, Germany) using a Mini Trans-Blot electrophoretic transfer cell (Bio-Rad) at 100 V for 30 min. The membrane was blocked with Tris-buffered saline with Tween 20 (TBST; 20 mM Tris, 150 mM NaCl, and 0.05% [v/v] Tween20, pH 7.6) containing 5% (w/v) non-fat milk powder at 20–22°C (room temperature [RT]) for 2 h. After washing thrice with TBST, the membrane was probed with mouse monoclonal anti-His antibodies (Dianova, Hamburg, Germany) at a 1:1,000 dilution in Signal Boost ImmunoReaction Enhancer solution I (cat. No. 407207; Merck KgaA, Darmstadt, Germany) at RT for 2 h. After washing, the membrane was probed with horseradish peroxidase (HRP)-conjugated donkey anti-mouse antibodies (Dianova) at 1:10,000 dilution at RT for 1 h. After a final wash with TBS, signals were detected via incubation with ECL chemiluminescence reagent and subsequent exposure of the membrane to a Kodak Biomax light X-ray film (VWR; Darmstadt, Germany).

### 2.6 Mouse immunization

Animal experiments were approved by Landesamt für Landwirtschaft, Lebensmittelsicherheit und Fischerei (Mecklenburg-Vorpommern, Germany; approval No. 7221.3-1-071/20-2) and performed in accordance with the German animal protection regulations.

Two groups of female BALB/c mice (6–8-weeks-old, n = 10 per group) were used for the immunization study (BIOSERV Analytik; Rostock, Germany). Each animal in the treatment group was injected subcutaneously with a physical mixture of partially purified plant-produced mZP2-3 and mIzumo1-3, containing approximately 5 µg of mZP2 and mIzumo1 peptides, respectively. Control group received similarly prepared extracts from pICH31120-infiltrated leaves. Mice were immunized four times at three-week intervals. All doses contained 10% Polygen (MVP Lab) as an adjuvant. Serum samples were collected prior to primary immunization and 20 days after each immunization to measure the antibody levels. Female BALB/c mice were mated with proven fertile BALB/c males of a similar age four weeks after the final immunization. Males were removed after two weeks, and the females were allowed to litter. Fertility was defined as the mean number of pups born.

### 2.7 Determination of antibody levels using enzyme-linked immunosorbent assay (ELISA)

Specific IgG responses in sera were determined using ELISA. Partially purified mZP2-3 and mIzumo1-3 were used to coat a 96-well plate. After incubation at RT for 2 h, the plates were washed thrice with phosphate-buffered saline (PBS) containing 0.05% Tween 20 and blocked with PBS containing 1% (w/v) BSA for 1 h at RT. After washing with PBST, diluted sera (1:25,000 in PBS) were added to the wells and plates were incubated for 2 h at RT. After washing with PBST, the plates were incubated with HRP-conjugated donkey anti-mouse secondary antibodies (Dianova) at a dilution of 1:2000 in PBS at 37°C for 1 h. After washing, the wells were incubated with tetramethylbenzidine substrate for 10 min in the dark, reaction was stopped using 250 mM H_2_SO_4_, and the OD at 450 nm was measured using a plate reader (Bio Tek; Bad Friedrichshall, Germany).

### 2.8 Histological examination

On day 135 of the experiment, mice were sacrificed. To monitor the effect of immunization on follicular development, ovaries were collected, fixed in 4% paraformaldehyde, and embedded with paraffin. Ovaries were sectioned at 5 µm, stained with hematoxylin and eosin, and observed under a microscope.

### 2.9 Immunofluorescence assay

Ovarian sections of immunized BALB/c and unimmunized wild mice were obtained from BIOSERV Analytik (Rostock, Germany). Ovarian sections were deparaffinized and subjected to antigen retrieval using sodium citrate buffer (10 mM sodium citrate, 0.05% Tween 20, pH 6.0). Sections were blocked with 10% goat serum blocking solution (Life Technologies, Frederick, Maryland, USA) at RT for 1 h, washed with PBS, and incubated with 1X mouse-on-mouse IgG blocking solution (Invitrogen, Thermo Fisher Scientific) at RT for 1 h. Sections of unimmunized wild mice were incubated with a 1:20 dilution of serum samples from vaccinated BALB/c mice and kept at 4°C overnight. Sections of immunized BALB/c mice were incubated with a blocking solution (without the addition of any serum samples), washed, and incubated with 10 µg/mL fluorescein isothiocyanate (FITC)-conjugated goat anti-mouse IgG secondary antibody (Invitrogen, Thermo Fisher Scientific) at 37°C for 1.5 h. After washing, slides were mounted with the DABCO mounting medium (25 mg/mL DABCO, 90% glycerol, and 10% PBS; pH 8.5) and observed under a fluorescence microscope.

### 2.10 Statistical analysis

Results are presented as the mean ± standard deviation. Experimental data were analyzed using IBM SPSS statistical software version 27. One-way analysis of variance and Student’s *t*-test were used to compare the means. Duncan’s test was used as a *post-hoc* test to measure the specific differences between the means. Statistical significance was set at *p* < 0.05.

## 3 Results

### 3.1 Transient expression of recombinant mZP2 and mIzumo1 peptides in *N. benthamiana* leaves

To determine the transient expression of peptides mZP2 (TDVRYKDDMYHFFCPAIQA) and mIzumo1 (LDCGERHIEVHRSEDLVLDCL) in *N. benthamiana*, codon-optimized constructs were cloned separately into the expression vector, pICH31120, of the MagnICON system. Expression cassettes were introduced separately into *N. benthamiana* leaves via agroinfiltration. Constructs mZP2-3 and mIzumo1-3 contained three repeated copies of antigenic peptides (**Figure 1**). A His-tag was added for the detection and purification of proteins.

**Figure 2:**
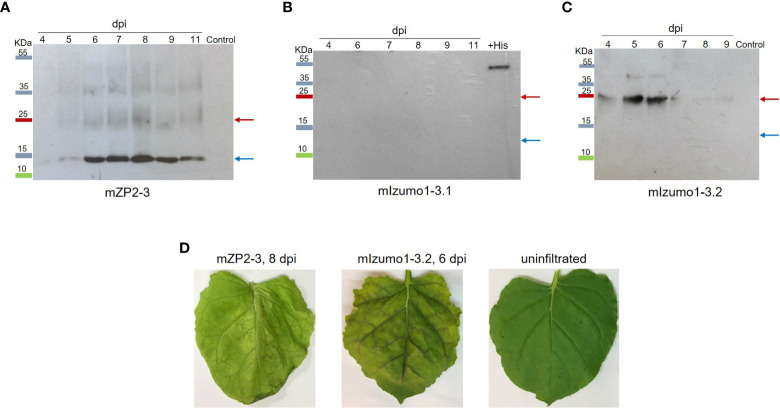
Expression of mZP2-3 and mIzumo1-3 in *Nicotiana benthamiana* leaves. Western blotting analysis of extracts from leaves infiltrated with mZP2-3 **(A)**, first mIzumo1-3 **(B)**, and second mIzumo1-3 **(C)** constructs at different dpi. Membranes were probed with a mouse anti-His antibody. Control, extract from pICH31120-infiltrated leaves; +His, a His-tagged protein as positive western blot control; dpi, days post-infiltration. Blue and red arrows indicate the expected size of the monomer and dimer forms of the proteins, respectively. **(D)** Phenotype of infiltrated leaves on optimal harvest day.

Infiltrated leaves were harvested over time, and the expression levels of mZP2-3 and mIzumo1-3 were determined using western blotting. A strong protein band with an expected size of approximately 13 kDa for monomeric proteins confirmed the expression of mZP2-3. Weak signals were detected at approximately 26 and 39 kDa, representing the dimeric and trimeric forms of the protein, respectively. Production of mZP2-3 was initiated on 4 dpi, increased until 8 dpi, and then decreased. In contrast, the control sample (pICH31120-infiltrated leaf extract) exhibited no signal (**Figure 2A**).

Plants infiltrated with the mIzumo1-3.1 construct, in which the His-tag was located in the C-terminal region, exhibited no protein band in the western blot using antibodies against His tag (**Figure 2B**). Therefore, a second construct (mIzumo1-3.2), with a His-tag at the N-terminus, was designed to express mIzumo1 in plants. Western blotting analysis of crude protein extracts from leaves infiltrated with mIzumo1-3.2 revealed a band of approximately 25 kDa corresponding to the dimer form of mIzumo1-3 protein (**Figure 2C**); therefore, mIzumo1-3.2 was used for subsequent experiments. The highest accumulation of mIzumo1-3 was detected on 5–6 dpi.

Infiltrated leaves developed a necrotic phenotype that was more severe in leaves expressing mIzumo1-3 than in those expressing mZP2-3 (**Figure 2D**). Optimal harvesting time points for mZP2-3 and mIzumo1-3 were determined to be 8 and 6 dpi, respectively, when the protein accumulation was high. Subsequently, severe leaf necrosis and wilting were observed along with a decrease in protein levels.

**Figure 3:**
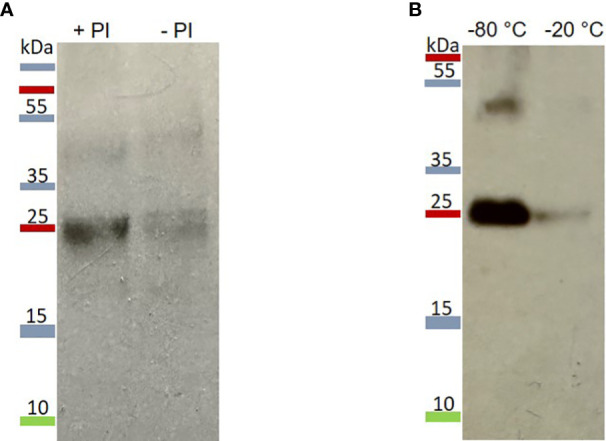
Stability of extracted mIzumo1-3. **(A)** mIzumo1-3 extracted from leaves with or without the addition of protease inhibitors. PI, protease inhibitor. **(B)** Detectable mIzumo1-3 after six weeks storage at –80 or –20°C. Membranes were probed with anti-His monoclonal antibodies.

After protein extraction, mIzumo1-3 showed low stability in the extract. The stability of mIzumo1-3 protein was increased after the addition of protease inhibitors (**Figure 3A**) and storage at –80°C (**Figure 3B**).

### 3.2 Purification of plant-produced mZP2-3 and mIzumo1-3

**Figure 4:**
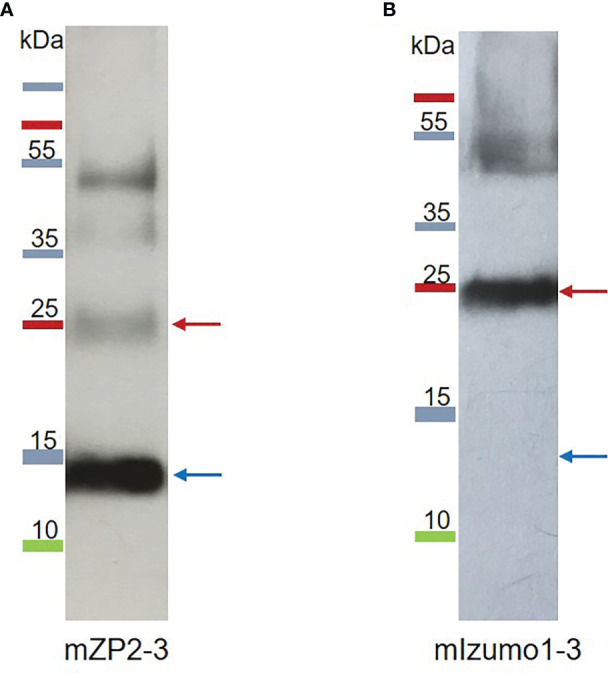
Western blotting analysis of partially purified plant-produced mZP2-3 **(A)**, and mIzumo1-3 **(B)** proteins. Proteins were purified from *N. benthamiana* leaves via Ni-NTA affinity chromatography, separated via sodium dodecyl sulfate-polyacrylamide gel electrophoresis (SDS-PAGE), and probed with anti-His monoclonal antibodies for western blotting. Blue and red arrows indicate the monomer and dimer forms of the proteins, respectively.

Recombinant mZP2-3 and mIzumo1-3 were partially purified from the crude plant extracts under non-denaturing conditions using Ni^2+^-charged column chromatography. Analysis of the partially purified mZP2-3 via western blotting revealed a dominant band of approximately 13 kDa and bands with molecular mass consistent with the monomeric and oligomeric forms of the antigenic construct, respectively (**Figure 4A**). In western blotting of the partially purified mIzumo1-3, a dominant band of approximately 25 kDa corresponding to the expected size of the dimer, and a band at the expected size of the tetramer form of mIzumo1-3 were detected, similar to those observed in the western blots of crude leaf extracts (**Figure 4B**). As the monomeric form of mIzumo1-3 was not detected via western blotting, a mass spectrometric analysis of the partially purified protein was performed, which confirmed the presence of mIzumo1-3 protein (**Supplementary Material, Figure S1**). ELISA revealed that the concentration of partially purified mZP2 was 29.3 µg/g dry weight (DW) and that of mIzumo1 was approximately 4.5 µg/gDW.

### 3.3 Immunogenicity of plant-expressed mZP2-3 and mIzumo1-3 in mice

**Figure 5:**
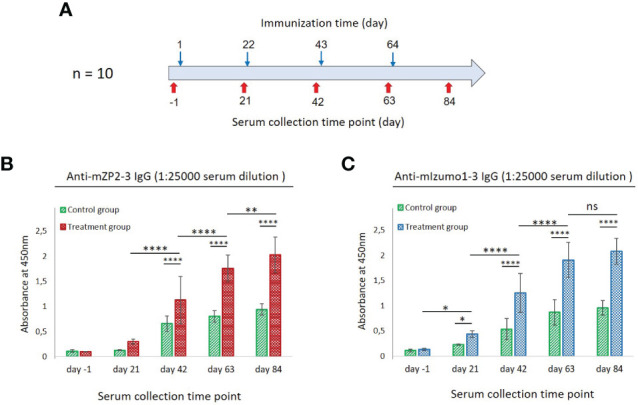
Antibody responses to mZP2-3 and mIzumo1-3 after subcutaneous immunization of mice. **(A)** Schematic representation of immunization and blood collection. Enzyme-linked immunosorbent assay (ELISA) for detection of anti-mZP2-3 **(B)** and anti-mIzumo1-3 **(C)** antibody responses in sera collected from the treatment (mice vaccinated with a mixture of plant-produced mZP2-3 and mIzumo1-3) and control (injected with the control plant extract) groups. Absorbance values are shown at 1:25,000 dilution of sera. Data are represented as mean ± standard deviation (SD). Absorbance was measured at 450 nm. *p < 0.05; **p < 0.01; ****p < 0.0001; ns, not significant.

To determine their immunogenicity, mZP2-3 and mIzumo1-3 were partially purified on a large scale and used in a prime-boost immunization assay. A group of 10 female BALB/c mice was immunized subcutaneously (four times at three-week intervals) with a mixture of equal amounts (5 µg) of plant-produced mZP2-3 and mIzumo1-3 antigens (**Figure 5A**). Mice in the negative control group were immunized with similarly prepared control plant extracts.

Humoral IgG antibody responses from individual mice were analyzed using ELISA at 1:25000 dilution of pre-immune and immune sera using plant-produced mZP2-3 or mIzumo1-3 as antigens. After primary immunization, no significant anti-mZP2-3 immune responses were detected in the sera of immunized mice compared to the pre-immune sera collected before primary immunization and that from the control group. However, second immunization induced an IgG antibody response that was significantly (*p* < 0.0001) higher than that in the primary dose and control group. Each subsequent immunization significantly increased the anti-mZP2-3 antibody response (**Figure 5B**).

Immunized mice exhibited a more potent anti-mIzumo1-3 antibody response than that observed at the pre-immune levels, even after primary vaccination with a physical mixture of recombinant proteins (**Figure 5C**). Immunization with the second and third doses significantly (*p <*0.0001) increased the antibody levels against mIzumo1-3. Antibody response continued to increase after the last immunization; however, it was not significant compared with that after the third dose (**Figure 5C**). Although some absorption was detected in the serum samples of the control group, it was significantly (*p* < 0.0001) lower than that in the serum samples of the antigen-vaccinated group (**Figures 5B, C**).

### 3.4 Effects of immunization on fertility and ovarian morphology

**Figure 6:**
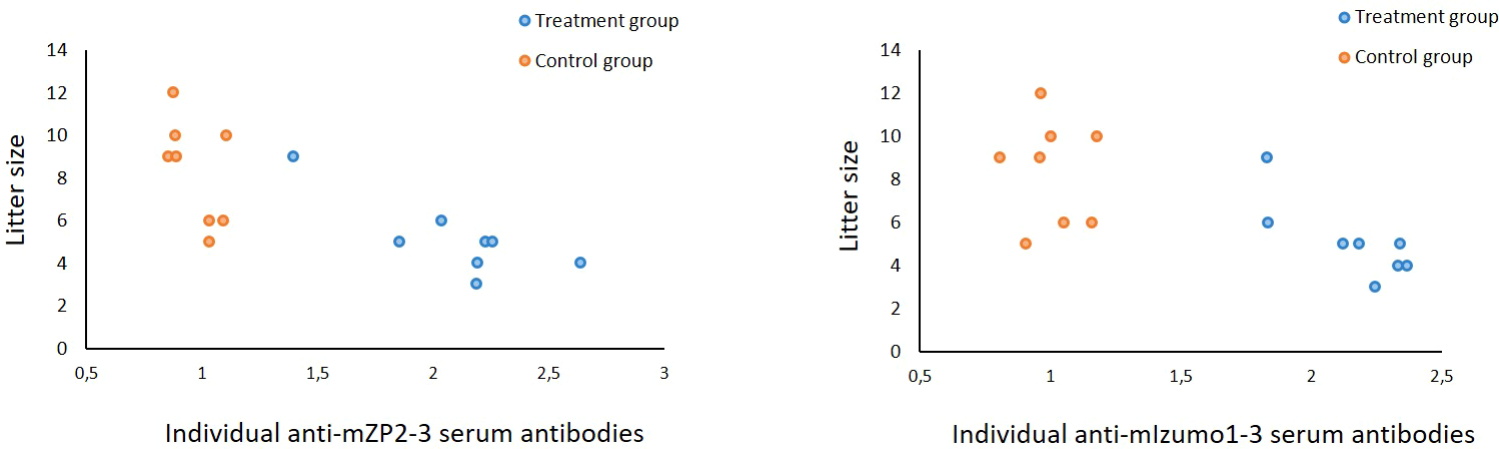
Correlation between anti-mZP2-3 and anti-mIzumo1-3 antibody levels and the litter size. Blue circles indicate IgG values in individuals in the group vaccinated with the mixture of mZP2-3 and mIzumo1-3. Orange circles indicate the IgG values in the control group. A significant negative correlation between the antibody levels and litter size was observed in the treatment group (*p* < 0.05).

Immunized female mice were mated with proven fertile male mice three weeks after the final vaccination and allowed to produce litters to determine the effect of immunization on mouse fertility. Mice in the group treated with mZP2-3 and mIzumo1-3 showed a 39% reduction in average fertility compared to that in the control group. Average litter size was reduced from 8.4 ± 0.86 (mean ± standard error of the mean) in the control group to 5.1 ± 0.64 in treatment group immunized with mixture of plant-produced mZP2-3 and mIzumo1-3 antigens. Although the difference in mean litter size between the groups was not significant, the number of pups born correlated with the level of individual antibodies against both mZP2-3 and mIzumo1-3 proteins (*p* < 0.05; **Figure 6**). Moreover, negative correlation between litter size and total antibody levels (anti-mZP2-3 + anti-mIzumo1-3) in individual mice was significant (*p* < 0.01; **Supplementary Material, Figure S2**).

**Figure 7:**
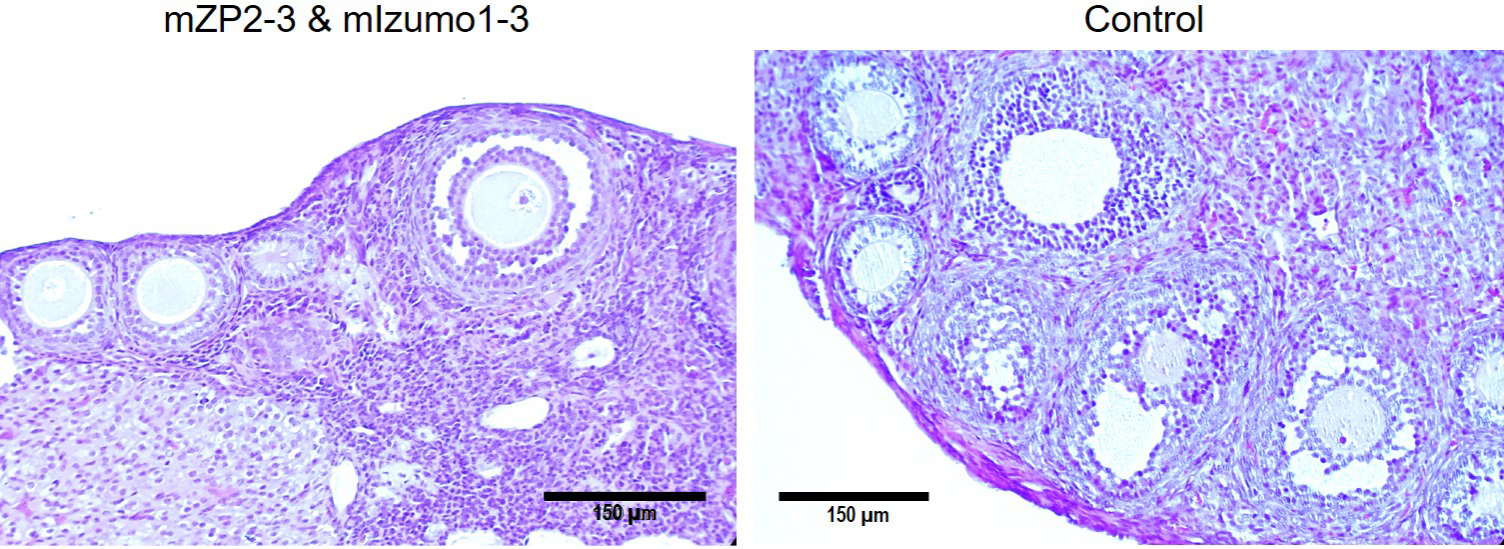
Ovarian histology of immunized BALB/c mice. Section from ovary of mouse immunized with the mixture of plant-produced mZP2-3 and mIzumo1-3 (left) exhibited normal growing follicles at various development stages similar to that from the control mouse immunized with the control plant extract (right). Ovarian sections were stained with hematoxylin and eosin. Scale bar: 150 µm.

To determine whether immunization with recombinant antigens negatively impacts the ovary, mouse ovaries were isolated for observation. Immunization with plant-produced antigens caused no oophoritis or damage to the ovaries as they appeared normal. Additionally, histological analysis of ovarian sections from immunized mice did not reveal any abnormal follicular development or follicular degeneration. **Figure 7** shows the normal follicles/oocytes at various stages of growth and development.

### 3.5 *In vivo* localization and *in vitro* binding of antibodies to native ZP

**Figure 8:**
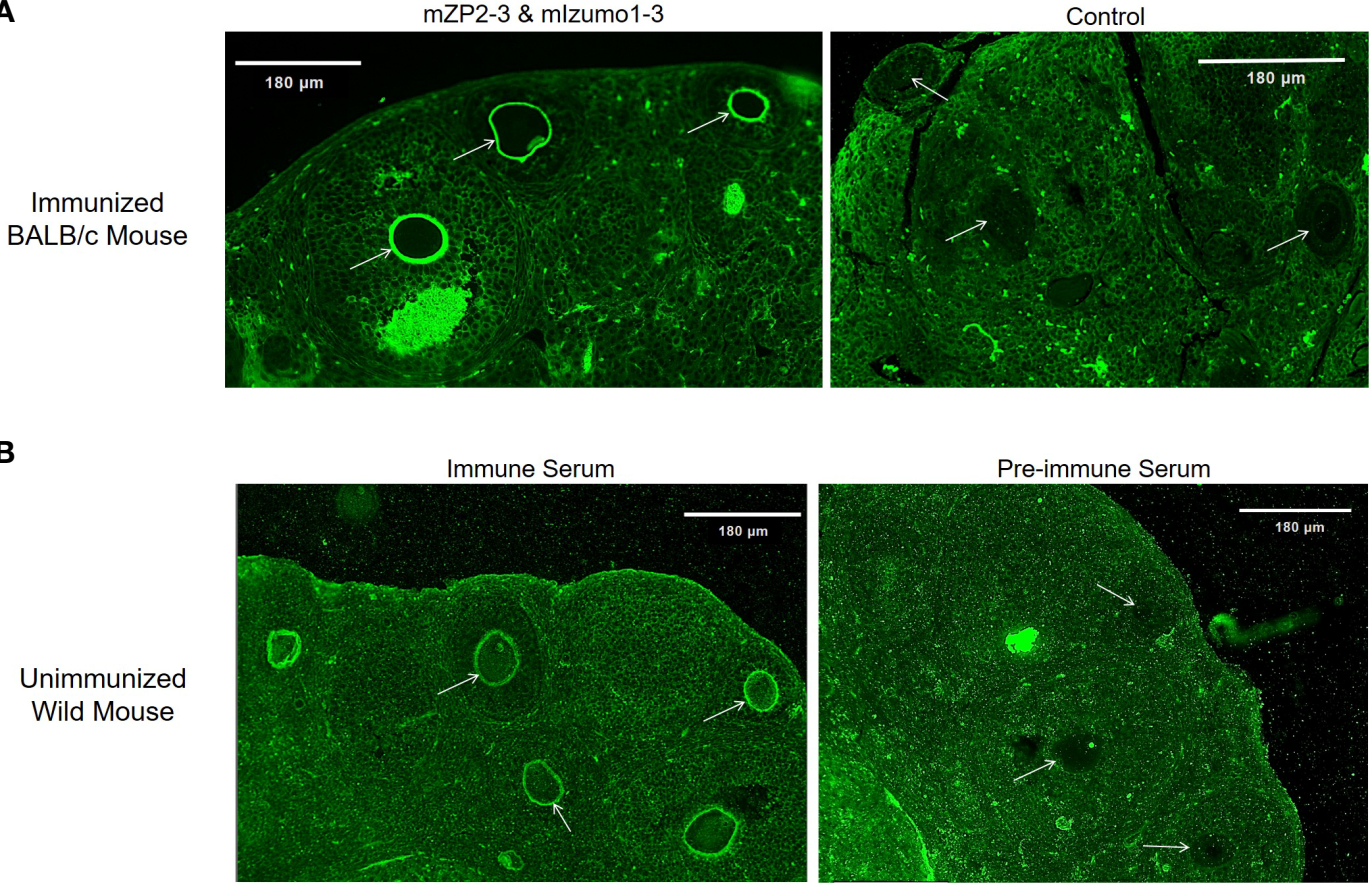
Reactivity of antibodies generated in immunized mice with zona pellucida (ZP). **(A)** *In vivo* binding of induced antibodies to the ovaries of immunized BALB/c mice. FITC-conjugated goat anti-mouse IgG detects induced antibodies bound to ZP in mice immunized with the physical mixture of plant-produced mZP2-3 and mIzumo1-3 (left), whereas no signal was observed on ZP of control mouse immunized with the control plant extract (right). **(B)** Indirect immunofluorescence assay for *in vitro* reactivity of BALB/c serum samples with wild mouse ZP. Ovarian sections were treated with 1:20 dilution of immune (left) and pre-immune (right) serum samples. Arrows indicate ZP. Scale bar: 180 µm.

Immunofluorescence assay was used to determine the presence of induced antibodies bound to ZP in immunized mice. In ovaries collected from mice immunized with a physical mixture of plant-produced mZP2-3 and mIzumo1-3, immunofluorescence was observed from ZP on the surface of oocytes, indicating that anti-mZP2 antibodies bind to ZP *in vivo*. Ovarian sections obtained from mice in the control group failed to show any signals from ZP (**Figure 8A**).

Immune serum samples obtained after the final injection of plant-produced antigens were used for their reactivity with the ovarian sections of wild mice in an indirect immunofluorescence assay. When serum antibodies produced in vaccinated BALB/c mice were applied to the ovarian sections from unvaccinated wild mice, fluorescence signals were observed around the oocyte, indicating the binding of antibodies to native ZP. Notably, ovarian sections treated with pre-immune serum did not show any antibody reactions with the ZP matrix (**Figure 8B**). Reactivity of the immune sera with spermatozoa was not evaluated as acrosome-reacted mouse sperms could not be obtained for this study.

## 4 Discussion

In this study, we aimed to investigate whether a combination of the small putative contraceptive peptides, mZP2 and mIzumo1, produced in *N. benthamiana* can reduce the fertility of immunized mice. Production of mouse-specific peptides is essential for their subsequent use as oral baits, which is the only practical way to immunize wild mouse populations. Plants are cost-effective and safe productions systems for the oral delivery of vaccines, without the need for any expensive purification steps (Daniell et al., 2009; Chan and Daniell, 2015). Moreover, plant-produced peptides can be bio-encapsulated in plant cells and protected from the gastro-intestinal environment that can limit the efficiency of free orally-delivered peptides (Kwon and Daniell, 2016; Pantazica et al., 2021). In addition, expression of contraceptive peptides in plants in fusion with highly stable proteins such as virus like particles can significantly improve their stability and immunogenicity to induce systemic and mucosal immunity in orally vaccinated mice (Ghasemian et al., 2023a).

Peptides were selected based on their immunogenicity, contraception capability (Sun, 1999; Naz, 2008; Wang et al., 2009), and potential mouse specificity (Hardy et al., 2008; Inoue et al., 2008; Xue et al., 2016). Previous studies have investigated the efficacies of synthetic peptides (Sun, 1999; Naz, 2008) or *Escherichia coli*-produced recombinant forms of peptides (Hardy et al., 2008; Wang et al., 2009) as contraceptive immunogens. Small peptides are generally unstable in plants (Kusnadi et al., 1997; Demain and Vaishnav, 2009). However, for the first time, we demonstrated the production of small peptides in sufficient amounts to induce antibody formation and reduce the fertility of BALB/c mice in this study. Moreover, we showed that the antibodies induced against plant-produced peptides can bind to wild-type mouse ovaries *in vitro*.

In accordance with our previous report that three repeats of mZP3 peptide significantly increased the antigen yield (Ghasemian et al., 2023b), the production of mZP2 and mIzumo1 in the form of three-peptide antigens resulted in sufficient amounts of the peptides in plants in this study. Our results confirmed the successful expression of the C-terminal His-tagged mZP2-3 construct. However, mIzumo1-3 protein was detectable using anti-His antibodies only when the His tag was placed at the N-terminus. Although localization of the His-tag is generally believed to have little or no effect on the structure and function of proteins, some studies have reported that the His tag and its position affect protein properties, such as production and stability (Mohanty and Wiener, 2004; Booth et al., 2018; Bulaon et al., 2020). Although mZP2-3 was detected at a molecular mass expected for the monomeric form, mIzumo1-3 was detected as a dimer, similar to previous reports demonstrating the ability of Izumo1 to form dimers that are stable and can survive SDS heat treatment (Ellerman et al., 2009; Inoue et al., 2013; Inoue et al., 2015). Moreover, His tags can mimic protein interactions and allow the protein to form higher-order oligomers (Mohanty and Wiener, 2004; Majorek et al., 2014).

Accumulation of mIzumo1-3 was significantly lower than that of mZP2-3 in infiltrated leaves. In addition to the rate of protein synthesis, the rate of protein degradation is an important factor affecting the final accumulation of recombinant proteins (Benchabane et al., 2009; Egelkrout et al., 2012). Here, we observed severe necrosis in leaves expressing mIzumo1-3 compared to leaves expressing mZP2-3. In response to infiltration, plants induce hypersensitive necrosis, which further induces the expression of proteases degrading the recombinant proteins (Pillay et al., 2014; Nosaki et al., 2021). Hence, the low accumulation of mIzumo1-3 in this study may be the result of degradation by plant proteases. Moreover, mIzumo1-3 was not stable after extraction in the absence of protease inhibitors, indicating the sensitivity of mIzumo1-3 to proteases released from plant organelles into the extract (Doran, 2006; Benchabane et al., 2009). Therefore, further optimization is needed to increase the stability and yield of mIzumo1 peptide in plants.

As equal amounts of mZP2 and mIzumo1 peptides needed to be administered and the volume of vaccine that could be injected into mice was limited, restricted amounts of the plant-produced peptides (5 μg of each in the mixture) were used in the immunization study. Compared with individual component epitopes, immunization with a combination of epitopes from different reproductive proteins involved in various steps of fertilization improves the immunogenicity and efficacy of vaccines (Hardy et al., 2004; Hardy et al., 2008; Choudhury et al., 2009). Here, we demonstrated the abilities of plant-produced mZP2-3 and mIzumo1-3 to induce significant levels of antibodies in immunized mice. Antibodies against mZP2-3 and mIzumo1-3 were detected in all immunized mice, although there was variation in antibody responses among individual mice, consistent with other reports (Hardy et al., 2008; Naz, 2008). In previous studies, anti-mZP2 and anti-mIzumo1 antibodies have been detected in mice after immunization with 20 µg of bacterial-produced mZP2 peptide (Hardy et al., 2008) or 75 µg of synthetic mIzumo1 peptide (Naz, 2008). However, in this study, 5 µg of plant-produced mZP2 and mIzumo1 was sufficiently immunogenic in mice. This might indicate the contribution of the production system in enhancing the immunogenicity of antigens (Schellekens, 2002). The responses detected in control mice may be due to the presence of antibodies against plant proteins co-purified with plant-produced immunogens and also present in ELISA detecting antigen (Naupu et al., 2020).

In this study, after the administration of plant-produced antigens, a 39% decrease in the mean number of pups was observed, which correlated with the levels of antibodies against both mZP2-3 and mIzumo1-3 antigens. *In vivo* binding of antibodies to the oocyte was detected in immunized animals. It is assumed that antibodies induced against the mZP2 peptide reached the target sites around the oocytes, preventing the spermatozoa from reaching the oocyte and efficiently disrupting fertilization (Duckworth et al., 2007). Immunity to these contraceptive peptides did not cause histological ovarian disruption or follicular depletion. Therefore, the observed decrease in litter size of vaccinated mice cannot be attributed to ovarian atrophy, oophoritis, or loss of normal follicles (Koyama et al., 2005; Lloyd et al., 2010). Although the reactivity of anti-mIzumo1 antibodies with sperms was not evaluated in this study, a significant correlation was observed between anti-mIzumo1 antibody levels and the number of pups born. The negative correlation between individual antibody responses and litter size suggests that higher antibody responses may lead to greater reduction in the number of pups. Compared with other studies, in which a significant reduction in fertility was achieved with higher concentrations of mZP2 and mIzumo1 (Hardy et al., 2008; Naz, 2008), a low dose (5 µg) of plant-produced mZP2 and mIzumo1 was sufficient to reduce the fertility of mice in this study. Immunization of mice with different concentrations of mIzumo1 peptide decreases the fertility in a dose-dependent manner (Naz, 2008; Wang et al., 2009; Naz, 2014). High doses of antigens induce sufficient levels of antibodies to significantly increase the contraceptive effect of vaccines (Pomering et al., 2002; Naz, 2008; Wang et al., 2009). These studies suggest that higher concentrations of plant-produced mZP2 and mIzumo1 may cause a greater reduction in fertility.

Reactivity of antisera from immunized mice with wild mouse oocytes indicated that immunization with plant-produced mZP2 generates antibodies reactive with the native ZP of wild mice. Although wild mouse sperms were not available for antibody reactivity assays, the correlation of anti-mIzumo1-3 antibodies with litter size suggested the contribution of mIzumo1 to the reduced fertility of immunized mice. Based on these data, we can infer that plants producing sufficient amounts of mZP2 and mIzumo1-3 may effectively reduce the fertility of the wild mouse populations via immunocontraception.

To the best of our knowledge, this is the first report on the production of the small contraceptive peptides, mZP2 and mIzumo1, using a plant production system. Our results demonstrated that mZP2 and mIzumo1 peptides can be expressed in *N. benthamiana*. We found that a low dose of plant-produced peptide antigens was immunogenic in mice and induced significant immune responses. Moreover, we observed a reduction in the number of pups and a negative correlation between litter size and the level of antibody response in mice vaccinated with plant-produced antigens. However, as only one antigen dose and a limited number of vaccinated animals were used in this study, further studies should use increasing doses of plant-produced peptides to induce higher titers of antibodies and investigate their effects on mouse fertility. Nevertheless, the findings of this study can be used to develop effective oral contraceptive vaccines to manage mouse populations.

## Data availability statement

The original contributions presented in the study are included in the article/**Supplementary Material**. Further inquiries can be directed to the corresponding author.

## Ethics statement

The animal study was reviewed and approved by Landesamt für Landwirtschaft, Lebensmittelsicherheit und Fischerei (Mecklenburg-Vorpommern, Germany; approval No. 7221.3-1-071/20-2).

## Author contributions

KG designed and performed the experiments, analyzed the data and wrote the manuscript. IB and JH supervised the work, gave scientific advice and revised the manuscript. JS supervised the histological and immunofluorescence studies and supported data interpretation. NK and RK carried out the animal experiment. SM performed mass spectrometric analyses. All authors contributed to the article and approved the manuscript.
