# Supplementary material for: Immunogenicity and contraceptive efficacy of plant-produced putative mouse-specific contraceptive peptides
Source: Front Plant Sci. 2023 Jun 28;14:1191640. doi: 10.3389/fpls.2023.1191640 (PMC10337994; doi:10.3389/fpls.2023.1191640)
Supplement: Supplementary file 1 [file DataSheet_1.docx]

Supplementary Material

**Immunogenicity and contraceptive efficacy of plant-produced putative mouse-specific contraceptive peptides**


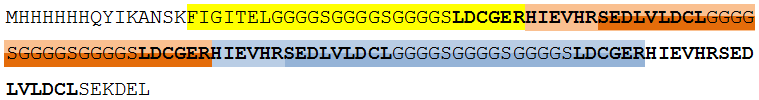


**Supplementary Figure 1.** Amino acid sequence of mIzumo1-3 protein. Three peptides identified by LC-MS/MS are colored in (i) yellow, (ii) light orange and light blue, (iii) darker orange and darker blue. The three copies of the mIzumo1 peptide are shown in bold letters.

**
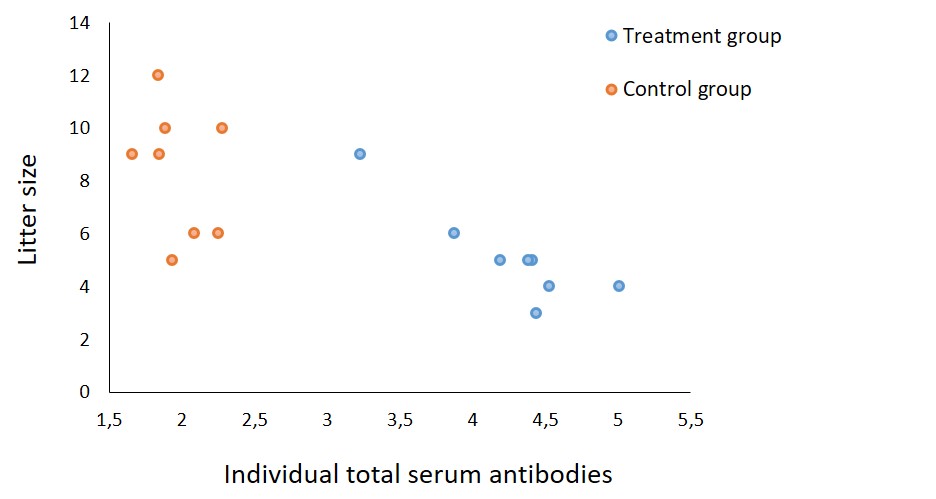
**

**Supplementary Figure 2.** Correlations between the total IgG (anti-mZP2-3 + anti-mIzumo1-3) antibody levels and the litter size. The blue circles indicate total IgG values from individuals in the group vaccinated with mixture of mZP2-3 and mIzumo1-3 and the orange circles indicate the values from control group. There was a significant negative correlation in the treatment group at the level of *p* < 0.01.
